# Physical Activity and Psychosocial Characteristics of the Peer Supporters in the PLAN-A Study—A Latent Class Analysis

**DOI:** 10.3390/ijerph17217980

**Published:** 2020-10-30

**Authors:** Ruth Salway, Simon J. Sebire, Byron Tibbitts, Emily Sanderson, Rebecca Kandiyali, Kate Willis, Stephanie J. MacNeill, Russell Jago

**Affiliations:** 1 Centre for Exercise, Nutrition & Health Sciences, School for Policy Studies, University of Bristol, 8 Priory Road, Bristol BS8 1TZ, UK; Simon.Sebire@bristol.ac.uk (S.J.S.); B.Tibbitts@bristol.ac.uk (B.T.); kate.willis@bristol.ac.uk (K.W.); Russ.Jago@bristol.ac.uk (R.J.); 2 Population Health Sciences, Bristol Medical School, University of Bristol, Bristol BS8 1TZ, UK; emily.sanderson@bristol.ac.uk (E.S.); rebecca.kandiyali@bristol.ac.uk (R.K.); stephanie.macneill@bristol.ac.uk (S.J.M.); 3 Bristol Randomised Trials Collaboration, Bristol Trials Centre, University of Bristol, Bristol BS8 1TZ, UK

**Keywords:** physical activity, peers, adolescent girls, latent class analysis

## Abstract

PLAN-A is a cluster randomised controlled trial of a peer-led physical activity intervention which uses peer supporters to increase the physical activity of 13–14-year-old girls in the UK. This paper uses latent class analysis to identify classes in the whole study population and investigate how those selected as peer supporters in PLAN-A were drawn from different social groups. We identified five classes of girls, based on psychosocial variables (self-esteem, physical activity self-efficacy, motivation, physical activity values among friends and peer support for physical activity (PA) and physical activity behaviour variables (average minutes of weekday MVPA, sedentary time and screen viewing). Peer supporters were similar to the whole study population in terms of overall demographics, but were drawn unequally from the five classes. In addition, there was considerable variation in the distribution of peer supporters between schools. The selection of peer supporters is an integral component of peer-led interventions and should be explored and linked to underlying theory to understand the characteristics of those recruited. However, demographic representativeness is not necessarily the aim, and simple reporting of overall demographic comparisons may mask important differences within subgroups.

## 1. Introduction

Physical activity is associated with lower levels of cardiometabolic risk factors and higher psychological well-being among children and adolescents [1,2,3,4,5]. The UK Chief Medical Officers recommend that all children and young people should engage in an average of at least 60 min of moderate-to-vigorous intensity physical activity (MVPA) per day across a week [6]. Several studies have shown that considerable proportions of adolescents do not meet this recommendation [7,8]. Moreover, physical activity levels decline with age and girls are less active than boys throughout childhood and adolescence [7,9]. Physical activity patterns track from adolescence into adulthood [10,11]. As such, there is a need to find ways to increase levels of physical activity amongst adolescent girls.

Systematic reviews have shown that the majority of physical activity interventions have either yielded no effect, small effect or effects in sub-groups [12,13]. While there have been a few notable exceptions [14,15], the overall pattern is of interventions that have not yielded the hypothesized impact. Most of these studies have focussed on changes to educational provision, or structural changes such as increased provision before or after school [16,17,18,19,20], therefore, alternative intervention approaches are needed.

Peers play a central role in adolescents’ physical activity through peer support, presence of peers during physical activity, peer norms, friendship quality and peer affiliation and there are consistent positive associations with physical activity/determinants of activity [21]. Peer-led interventions have been used to target smoking, asthma, alcohol consumption and drug use [22,23,24]. Most of these programs have been delivered in secondary education and trained peer supporters to influence other pupils through information provision, skill development and creation of new social norms. We have developed a peer-led physical activity intervention called PLAN-A aimed at increasing adolescent girls’ physical activity levels, a group whose physical activity levels are generally low and who face specific barriers to being active. PLAN-A was initially developed for Year 8 girls and then changed to Year 9 girls to comply with changes to the consent process that were required after the introduction of new General Data Protection Regulation (GDPR). Our formative work from a feasibility trial has shown that the PLAN-A intervention is acceptable and feasible with evidence of promise to impact on levels of physical activity [25,26]. The intervention is based on Diffusion of Innovations (DOI) Theory [27] and postulates that those who are identified by their peers as influential can work as agents of change by diffusing physical activity messages through their peer group. Peer supporters are therefore selected by a peer nomination process and then provided with 2 days (plus a 1-day booster session) of training, out of school, in the importance of physical activity, barriers faced by adolescent girls, and strategies to encourage and influence the physical activity of their peers through informal diffusion and facilitating behaviour change. Central to the intervention is the selection of the influential peer supporters.

Although peer-based interventions have become popular within the public health field, it is unclear what the key or most desirable characteristics of peer supporters are. The majority of peer-led interventions provide no description of selected peer supporters, and those that do typically only compare demographics of peer supporters to the study population. However, demographic representativeness is not necessarily the aim of peer selection. Some authors advocate selecting on the basis of peer supporter personal characteristics such as being experienced in the topic [28], or credible and trustworthy [29], while others emphasise network characteristics such as being popular or well-connected within peer networks [30,31,32]. Such criteria may distinguish peer supporters from the rest of the cohort and could mean that they differ from them in other ways, including demographically, which would mean demographic representativeness could obstruct selection of the ‘best’ peer supporters. In fact, some argue that interventions should explore engaging more of the target population as peer supporters [28], as similarities increase the persuasiveness of peer messaging [33], although it is important that peer supporters are credible [34]. From a DOI perspective, peer supporters should be the early adopters of the target behaviour, who are willing to embrace new concepts and act as opinion leaders and role models whose actions create new norms and inspire uptake of the behaviour amongst their peers [35]. While it is not clear how to easily identify the early adopters, interventions that apply the DOI theory seek to recruit peer supporters who are ‘influential’ within their networks and who are willing and motivated change agents. The ASSIST study (A stop smoking in schools trial) and PLAN-A, both based on this model, use a series of questions designed to identify influential peers using characteristics such as leadership, being respected, and being trusted. [22,36]. Evaluation of the ASSIST peer supporters focused on the extent to which peer supporters were drawn from a diversity of different social groups and their location within their diffusion network and found that peer supporters were drawn from a range of different social clusters, although 40–50% of clusters had no peer supporters [32]. However, whilst this peer nomination technique should lead to the selection of young people who are good leaders, respected, and trusted, the profiles or characteristics of the peer supporters on other variables relevant to the behaviour that they are trying to influence (e.g., physical activity) has not been studied in depth [37] but may be critical to the success of peer-led interventions.

This paper will explore the characteristics of those selected as peer supporters in PLAN-A in all schools blinded to subsequent allocation to intervention arm. We will use latent class analysis, which uses the observed data to identify underlying classes of similar participants, to identify classes in the whole study population, which will allow us to investigate how peer supporters are drawn from cohort profiles based on other characteristics related to physical activity behaviour.

## 2. Materials and Methods

Peer-Led physical Activity iNtervention for Adolescent girls (PLAN-A) is a cluster randomised controlled trial of a peer-led intervention to increase adolescent girls’ physical activity. This paper uses data from the full trial [38] following the success of the earlier feasibility trial [25]. The study was registered with ISRCTN (registration number ISRCTN14539759) and ethical approval was obtained from the School for Policy Studies ethics and research committee at the University of Bristol (REF—SPSREC17-18.C22). All girls in Year 9 (aged 13–14) from 20 participating schools in Southwest England were invited to take part. Schools that expressed an interest were invited to take part, with baseline data collected between October 2018 and February 2019. In the 20 schools there was considerable range in class size from 31 to 142, and variation in socio-economic background with schools drawn from all five quintiles of the Index of Multiple Deprivation (http://data.gov.uk/dataset/index-of-multiple-deprivation). Four of the schools were in a rural county 100 miles from Bristol with the remainder within the greater Bristol region. The study applied a dual parent and child consent process, with both pupil opt-in and parent opt-out consent received for all participants. The trial was randomised at school level to receive either the peer supporter training intervention or not. In total, 1558 girls consented to take part (85%) and baseline data on psychosocial variables and accelerometer-measured physical activity were collected for all girls prior to the selection of peer supporters. An additional 54 girls participated in the peer nomination even though they did not consent to take part in the study or contribute baseline data; as the process was anonymous their nominations cannot be removed from the nomination data. Thus peer supporters are identified on the basis of 1612 nominations, but the individual data used in this analysis is from all 1558 girls who consented to the PLAN-A trial, regardless of subsequent trial arm allocation. This paper is an exploratory analysis of baseline data collected before the intervention, to describe the characteristics of those selected as peer supporters and is blind to intervention status. While all girls in the year group were eligible for nomination, regardless of if they took part, we analyse only those nominated among the 1558 participants.

### 2.1. Peer Supporters

All girls were asked in an anonymous questionnaire to identify influential girls in their year by nominating up to five names for each of the following questions: ‘who do you respect?’, ‘who are good leaders in sports or other group activities?’, ‘who do you trust?’ and ‘who do you look up to?’. Any nominations to people outside the school year group or not at their school were removed. Students received just one vote per questionnaire for a nomination in any question, (i.e., if a student was nominated for multiple questions, they received just one vote). Votes were tallied, and following the approach of the ASSIST study [22], a cut-off number of nominations was identified for each school representing the highest-scoring 18% (those with the most nominations) of students. All girls in the school who received at least the cut-off number of votes were identified as potential peer supporters. In this paper we define ‘peer supporters’ to mean potential peer supporters from all schools regardless of subsequent allocation of schools to intervention or control trial arms or inclusion of the nominated students in the peer supporter training; for brevity we refer to these individuals as peer supporters.

### 2.2. Psychosocial Variables

Participants in the trial completed a questionnaire to assess the following psychosocial variables: self-esteem (Self Description Questionnaire II [39]), physical activity self-efficacy (Physical Activity Self-Efficacy Scale [40]), autonomous motivation, controlled motivation and amotivation (the Behavioural Regulation in Exercise Questionnaire (BREQ-2) [41]) and prevalence, perceived acceptance and perceived importance of physical activity among friends (Peer Norms Scale [42]). In addition, we asked six questions about peer support and encouragement for physical activity: *has anyone engaged in PA with you; invited you to engage in PA; commented positively on your performance; encouraged, watched or talked about PA with you*. These were averaged to provide a measure of peer support for physical activity.

Within self-determination theory [43] motivation types are ordered on a continuum with autonomous motivation (enjoyment, identity and value) as high quality and positively associated with physical activity and positive cognitive and affective outcomes [44] and controlled motivation (guilt and external rewards/pressures) as poorer quality and often short-lived, associated with negative psychosocial outcomes and fragile to challenge. A lack of either autonomous or controlled forms of motivation is classed as amotivation [45]. Amotivation was measured as the average of four questions on a 5-point scale, but was heavily skewed, and over half the girls (58%) had a zero score, corresponding to answering ‘0 - not true for me’ for all four questions. It was thus not possible to treat amotivation as a continuous normally distributed variable in the latent class analysis. Based on the data, we created a binary categorical variable for amotivation, with a value of ≤0.25 representing very low amotivation (where girls answered ‘not true for me’ for at least three questions, and no more than 1 for the last question) and a value of >0.25 representing comparatively high amotivation. Similarly, the three peer norm scale variables, representing values, attitudes and behaviours about physical activity among friends, were based on an average of two questions each on a 3-point scale, and so not continuous. For each of these we created binary categorical variables of low (0–2) versus high (3–6), with the cut-off based on the midpoint.

### 2.3. Physical Activity Measures

All participants wore an ActiGraph wGT3X-BT accelerometer (Actigraph LLC, Pensacola, FL, USA) for seven consecutive days. The accelerometer data files were downloaded using Actilife v6 (Actigraph LLC, Pensacola, FL, USA) and processed using Stata v15 ((StataCorp LLC, College Station, TX, USA) [46]. For each participant the accelerometer data were included if they provided two or more days of valid data. A valid day was defined as at least 500 min of data between 6 a.m. and 12 p.m., after excluding intervals of ≥60 min of zero counts. Raw data were collapsed into 10s intervals and characterised as sedentary or MVPA using Evenson population-specific cut points [47]. The average number of MVPA and sedentary minutes per weekday were derived for each participant, and whether they met the guidelines of an average of at least 60 min of MVPA per day across the whole week (including both weekdays and weekends).

### 2.4. Other Variables

Girls were additionally asked about the number of hours they typically spent engaging in specific screen-viewing behaviours (TV, computer, phone and games consoles) on weekdays; these were combined to estimate the total number of minutes spent on any type of screen-viewing on weekdays. We additionally asked about ethnicity and used two measures of socio-economic position: receipt of free school meals and the Family Affluence Scale [48] (on a scale between 0 and 9 with higher values indicating a more affluent household), used as a proxy for family-level socio-economic position.

### 2.5. Statistical Analysis

We reported missing data and descriptive summaries for the psychosocial (self-esteem, physical activity self-efficacy, motivation, physical activity values among friends and peer support for physical activity), physical activity behaviour (average minutes of weekday MVPA, sedentary time and screen viewing) and demographic variables (family affluence scale, receipt of free school meals and ethnicity) for the whole study population, and the peer supporters.

Latent class analysis is a latent variable model that uses observed variables to classify participants with similar patterns of measured characteristics into a set of mutually exclusive latent (unobserved) classes [49,50,51], similar to cluster analysis. It is a data-driven approach that uses observed data to determine clusters (latent classes) of girls who share similar characteristics. The observed variables may be categorical or continuous; in this analysis we used a mixture of categorical and continuous variables. Latent class analysis estimates probabilities of class membership for participants, rather than assigning a single class, which enables class-membership uncertainty to be appropriately modelled.

We fitted latent class models for all girls in the study, using the psychosocial variables (self-esteem, physical activity self-efficacy, motivation, physical activity values among friends and peer support for PA) and physical activity behaviour variables (average minutes of weekday MVPA, sedentary time and screen viewing). We allowed a common covariance between MVPA minutes and sedentary time across all classes since the variables are correlated in the way they have been derived (total PA is equal to the sum of MVPA, sedentary and light physical activity). In terms of model structure, all other covariates were assumed to be independent conditional on class membership. As described above, we used categorical versions of amotivation and peer norm scales. The remaining variables were treated as normally-distributed variables, rescaled to the interval [0, 1], with variances that differed between each other and between classes. We explored different specifications for the variance to assess the sensitivity of the final classes to these assumptions, and additionally explored sensitivity to the categorisation of amotivation and peer norm scales.

We fitted models for 2–10 classes, and reported the Bayesian Information Criterion (BIC) [52], where lower values indicate better model fit, and the Lo-Mendell-Rubin (LMR) [53] and bootstrapped likelihood ratio tests (BLRT) [54] which both compare the current model to one with one fewer classes. While there is no single criterion for choosing the optimal number of classes [55], these criteria have been shown to perform well at identifying an appropriate number of classes. We also reported the relative entropy, an overall measure of classification on a scale of 0 (random) to 1 (perfect classification) [56], and the smallest class size (to identify problematic models with very small class sizes). In determining the final number of classes, we used a mixture of the statistical criteria, parsimony and interpretability. Once the classes had been identified we estimated the proportion of each group who were selected as peer supporters, with confidence intervals using the Bolck, Croon, and Hagenaars (BCH) method [57,58] which includes the classification error and is robust to assumption violations. We also described differences in the class distribution between schools, based on most likely class membership.

We used full maximum likelihood, which uses available information from all participants and handles missing data within the analysis model. This has been shown to produce unbiased parameter estimates and standard errors in structural equation models when data are missing at random [59]. All analysis was performed using Mplus v8.4 (Muthén & Muthén, Los Angeles, CA, USA) [60] with standard errors adjusted to take account of the clustering within schools, and was in addition to the main proposed study analyses.

## 3. Results

Of the 1558 girls who consented to the PLAN-A trial, all provided questionnaire data and 96% of girls provided at least 2 days of valid overall physical activity data at baseline. Missing data for the variables used in the latent class analysis was low (Table S1), with 0–4% of psychosocial variables and 8% of physical activity measures missing for both peer supporters and the sample as a whole. The peer supporters represented 23% of the cohort and were similar to the whole study sample in terms of demographics and physical activity, but they had slightly higher scores for self-esteem, physical activity self-efficacy, autonomous motivation and value of physical activity among friends (Table 1).

Table S2 compares indicators of model fit and classification for 2–8 classes; the models for 9 and 10 classes were unstable and so we did not investigate these further. As there is no clear way to determine an optimal number of classes, we used the BIC, LMR and BLRT to identify a subset of preferred models, and then investigated the resulting classes in more detail. Models with 6–8 classes had the lowest values of BIC, indicating better fit, although differences between them were small. The LMR tests (testing the null hypothesis that each model was no better than that with one fewer classes) indicated that the 4-class model was preferred, while the BLRT rejected the null hypothesis for all models we explored, always preferring models with more classes. We investigated models with 4–7 classes in more detail by considering the class definitions and interpretability. The key difference between the 5 and 6-class models was the splitting of an ‘average’ class into two with slightly higher and lower values, but without any distinct differences between them, with the 7-class model splitting this group still further. The 4-class model, preferred by the LMR test, did not distinguish between different types of motivation in the low confidence and low PA value classes, which we considered to be important in the current context. Thus, we chose the 5-class model, as it best combined simplicity and classes with distinct and relevant features.

The 5-class model had good classification properties (relative entropy of 0.750), good certainty about class membership (average classification probabilities were over 80% for all classes) and no small classes (smallest class 12%). We explored several alternative model specifications, including different variance structures and categorisation of peer norm variables, and found similar classes, suggesting low sensitivity to variance and model assumptions. We also looked at the categorisation of the amotivation and peer norm scales and found that using slightly different cut-offs produced very similar classes and distribution of classes.

**Figure 1:**
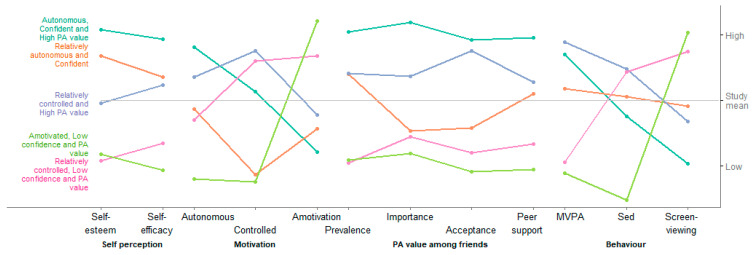
Characteristics of the five classes. Vertical axis is centred on the overall study mean for each variable and scaled to the range of the classes. MVPA = mins of moderate-to-vigorous intensity physical activity; Sed = mins of sedentary time.

The five classes are shown in Figure 1; full details are given in Table S3. The *Autonomous, Confident and High PA value* class (19%) has high levels of autonomous relative to controlled motivation, very high levels of self-esteem and self-efficacy, and high value of physical activity among friends. The *Relatively autonomous and Confident* class (18%) have moderate autonomous motivation and very low controlled motivation, high levels of self-esteem and physical activity self-efficacy, and average to low value of physical activity among friends. The *Relatively controlled and High PA value* class (28%) has moderate-to-high quantity of motivation, but of mixed quality, consisting of higher controlled versus autonomous motivation, average self-esteem and self-efficacy and average to high value of physical activity among friends. The *Relatively controlled, Low confidence and PA value* class (23%) has lower quantity of motivation, with high levels of controlled relative to autonomous motivation and high amotivation, low self-esteem and physical activity self-efficacy and low value of physical activity among friends. The final *Amotivated, Low confidence and PA value* class (12%) has low autonomous and controlled motivation and high amotivation, low levels of self-esteem and physical activity self-efficacy and low value of physical activity among friends. Physical activity varied more within classes than between classes, with the two classes characterised by low confidence and low PA value having lower levels of MVPA on average, and the classes characterised by higher autonomous motivation and PA value having slightly higher levels. Screen-viewing was lowest in the *Autonomous, Confident and High PA value* class, and very high in the two classes characterised by low confidence and low PA value.

**Figure 2:**
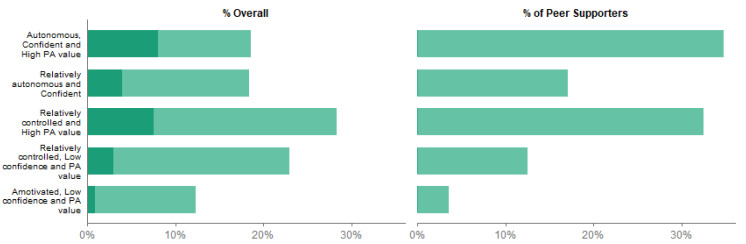
Prevalence of the five classes overall (left), and among peer supporters. Left bar plot also shows the proportion of each class who were selected as peer supporters (darker shaded).

We estimated the proportion of girls in each class who were nominated as peer supporters. Figure 2 shows the distribution of all girls across the five classes (left) compared to the distribution of peer supporters in the five classes (right), and additionally the proportion of each class who were chosen as peer supporters (shaded area, left). While nearly half of the sample (47%) are in the mixed and autonomous motivation classes, two thirds of peer supporters (67%) are in these classes. The peer supporters are predominantly from the mixed and autonomous motivation, with only 7% from the *Amotivated, Low confidence and PA value* class but 42% from the *Autonomous, Confident and High PA value* class. In addition, there were differences in socioeconomic factors between latent classes (Table S3), with those in the *Autonomous, Confident and High PA value* class having a higher average family affluence scale than the *Amotivated, Low confidence and PA value* class (7.5 compared to 6.1), and substantially fewer pupils receiving free school meals (5% compared to 21%). The distribution of classes, and peer supporters within those classes, differed substantially between schools (Figure S1). Although there was considerable variation in school sample sizes (between 31 and 142), around half of schools (both large and small) had no peer supporters from the *Amotivated, Low confidence and PA value* class and a fifth had none from the *Relatively controlled, Low confidence and PA value* class.

## 4. Discussion

We found that in PLAN-A, peer supporters were more likely to be characterised by autonomous motivation and high self-esteem than their broader peer group. This is unsurprising as the peer nomination process was designed to identify influential girls who might be expected to differ from their peers. However, peer supporters differed markedly from the whole study sample despite being similar in terms of simple demographics such as ethnicity and socio-economic status. Moreover, our latent class analysis provides a more detailed exploration of the characteristics of peer supporters and a deeper understanding of how they differ from their peers than that given by simple overall comparisons, which may miss potentially important differences. Thus, peer-led interventions should not rely solely on the basis of demographic comparisons to understand the characteristics of peer supporters and explore any differences with their peers, as this can mask important variation within subgroups. It is therefore important that future studies also examine the broader characteristics of the peer supporters and how they are identified.

The classes grouped girls according to self-esteem, physical activity self-efficacy, motivation, physical activity values and support among friends and physical activity behaviour. As a result, we do not have details of friendship networks to be able to determine how connected the peer supporters were to their peers, or to determine if there were clusters without representation who might remain isolated. Classes more strongly reflected the psychosocial measures than physical activity behaviour, and so peer supporters were predominantly girls with autonomous motivation (that is motivation for being active for enjoyment, personal value or alignment with their broader sense of self) and positive attitudes about physical activity.

While on average girls in these classes engage in more MVPA and less screen-viewing than those in other classes, the large variability means that it is not possible to draw conclusions about the physical activity of peer supporter. While it could be that influential girls were more likely to belong to the *Autonomous, Confident and High PA value* class, it could also be the case that the peer nomination process was more likely to identify confident and motivated girls. Although the peer nomination questions were intended to be general and not specifically focused on physical activity, biases may have crept in. For example, information and consent procedures included reference to activity monitors and occurred prior to nomination, and although a standardised script was followed when the peer nomination process was introduced to participants, it may be that verbal cues to physical activity were made beforehand (e.g., from teachers who may have made generic reference to ‘the sport study’ or ‘the physical activity project’). Furthermore, the question ‘who are good leaders in sports or other group activities?’ specifically mentions sport as a criterion.

The DOI theory [27] is based on the idea that peer supporters will talk with their friends, model active behaviours and positive attitudes towards physical activity, and create new norms and that these attitudes and behaviours will diffuse throughout their close network. In PLAN-A the nomination process was anonymous (in terms of the person making the nominations) so that young people are free to nominate any of their peer group, without fear of disclosure or negative implications on their friendships. However, the latent class analysis used questions about the prevalence, importance, acceptability and general support for physical activity among their friends, and so the classes do reflect some social aspects of friendship groups, particularly with regard to attitudes towards physical activity. While the distribution of peer supporters is unequal, there are representatives in all five classes overall, so the nomination process did capture girls from groups with diverse psychosocial attitudes towards physical activity. However, the distribution of the peer supporters varied substantially between schools, with some schools having no peer supporters in the *Amotivated, Low confidence and PA value* class, despite having fair proportions of year 9 girls within this class. These classes are data-driven rather than genuine observed cliques (which the girls themselves might recognize), and we should not assume that girls in different classes do not interact with each other. However, a social network analysis of the ASSIST study [32] found nearly half of clusters contained no peer supporters, and so this does highlight the importance of reaching girls who may fall outside the influence networks of the peer supporters. It is therefore particularly important that the peer supporters can understand the barriers that other girls might face to being active and use peer support strategies which take these into account. Indeed, empathy, support and supporting peers’ autonomous motivation (i.e., helping peers to find/try a physical activity that they value or enjoy—in and of itself, or for reasons such as spending time with friends) is a fundamental foundation of the PLAN-A peer-supporter training. Our feasibility study process evaluation demonstrated that girls identified the need for and were adept at using subtle, empathic and autonomy-supportive peer support techniques [61].

There was substantial variation between schools, both in terms of the distribution of classes and of peer supporters within those classes, although sample sizes were small in some schools. For example, in some schools, the dominant profiles were characterised by amotivation, controlled motivation and low friend PA values, whereas others were dominated by profiles of higher quality autonomous motivation, greater confidence and higher friend PA value. This is not surprising, as the schools in the study are socio-economically diverse, in terms of deprivation and urban/rural location, and the classes differed in socio-economic factors such as family affluence scale and those receiving free school meals (Table S3 in Supplementary Material). However, the large between-school variation may also reflect other school differences, both PA-related factors (such as the importance placed on PA, common attitudes towards and provision for girls being active) and non-PA factors (such as socio-economic factors and social groupings). Further research is needed to explore the importance of the wider school context, but this could play an important role in the understanding of peer-led interventions, where social structure is central to the theory underpinning the intervention. Currently, peer-led interventions focus on individual effects. Previous work has shown that friends play an important role in physical activity, with similar activity levels between children and their close friends, and evidence that friends influence each other’s physical activity among adolescents [62,63,64]. In addition, the importance of friendship networks increases as children age, with around 20% of the variability in MVPA attributable to friendship networks for girls at age 11 [65]. Thus, there may be mediating effects at school and/or network level as well as at an individual level. Analysis of such effects is likely to be complex and it may be difficult to estimate school or network effects in studies that are powered to detect individual intervention effects.

To better understand whether peer-led interventions have successfully recruited their target group, peer-led interventions need to be clear about who they intend to recruit. This can be helped by linking peer supporter characteristics to an underlying theory or theories of how peers are expected to influence their friends’ behaviour. It would also be advantageous for peer nomination, or selection techniques to be validated using other indicators of the desired peer-supporter characteristic (e.g., being influential, however this is defined). Statistical techniques, such as latent class analysis or social network analysis, may also provide insights into identifying influential actors, validating the peer nomination process or exploring the characteristics of those recruited. In addition, future interventions could explore the social dynamics of the school and peer social networks rather than just concentrate on individual effects. It is important to recognize that peer-led interventions are set within the context of friendship groups which often change during secondary school. As such, it may be the case that these programs need to be flexible to take account of these changes, for example, by repeating the friendship nomination process to assess changes.

### Strengths and Limitations

The major strength of this paper is the novel application of latent class analysis to compare the characteristics of peer supporters and fellow students within the school year group. The study is also strengthened by the low level of missing data and the use of a range of psychosocial variables and accelerometer-assessed physical activity. The current study does, however, have several limitations that need to be recognised. First, the study was conducted in the Southwest of England with only Year 9 girls and whilst providing an in-depth insight into this group, the ability to generalise to other areas, ages and boys is limited. There is also a lack of other contextual data such as information on sport participation or membership of social networks which may have been helpful.

## 5. Conclusions

In this paper, using psychosocial and behavioural variables related to PA we identified five latent classes of girls participating in the PLAN-A study, and found that the distribution of peer supporters across the classes differed notably from the whole study population. However, this was not evident from a comparison of overall demographics. The selection of peer supporters is an integral component of peer-led interventions and may play a critical role in their success. It is therefore essential that peer-led interventions are clear about who they intend to recruit and that the selection and characteristics of peer supporters are explored in detail and linked to underlying theory. Simple reporting of overall demographic comparisons may mask important within-subgroup differences.

## Figures and Tables

**Table 1:** Participant characteristics for whole sample and peer supporters.

Characteristics	All (N = 1558) mean (SD)	Peer Supporters (N = 357) mean (SD)
**Demographics**		
Family Affluence Scale	6.8 (1.8)	7.1 (1.7)
Free school meals	11%	8%
Ethnicity		
White	89%	89%
Non-white	11%	11%
**Self perception**		
Self-esteem ^1^	0.67 (0.22)	0.74 (0.20)
Physical activity self-efficacy ^1^	0.69 (0.22)	0.77 (0.19)
**Motivation**		
Autonomous ^1^	0.62 (0.25)	0.75 (0.22)
Controlled ^1^	0.33 (0.20)	0.34 (0.19)
Amotivation ^1^	0.11 (0.17)	0.06 (0.14)
**PA among friends**		
Prevalence ^1^	0.49 (0.29)	0.54 (0.20)
Importance ^1^	0.49 (0.24)	0.55 (0.31)
Acceptance ^1^	0.40 (0.22)	0.44 (0.31)
PA peer support ^1^	0.48 (0.22)	0.53 (0.20)
**PA behaviours**		
Average weekday MVPA (mins)	51 (20)	54 (20)
Average weekday sedentary (mins)	591 (93)	587 (86)
Average weekday screen-viewing (mins)	390 (241)	354 (208)
% meeting PA guidelines	22%	27%

^1^ Scaled to values between 0 and 1, for ease of comparison. MVPA = mins of moderate-to-vigorous intensity physical activity, PA: physical activity.

## Data Availability

The datasets generated during the current study are not publicly available due as the project is ongoing and data are not ready for archiving. We will consider reasonable requests for access to the data after the project is complete in 2021.

## Supplementary Materials

The following are available online at https://www.mdpi.com/1660-4601/17/21/7980/s1, Table S1: Missing data for the whole study and among peer supporters; Table S2: Model fit and classification for models with 2–10 classes; Table S3: Item response probabilities and means for final 5-class model; Figure S1: Distribution of classes and peer supporters between schools; Mplus code.
