# Supplementary material for: Physical Activity and Psychosocial Characteristics of the Peer Supporters in the PLAN-A Study—A Latent Class Analysis
Source: Int J Environ Res Public Health. 2020 Oct 30;17(21):7980. doi: 10.3390/ijerph17217980 (PMC7663228; doi:10.3390/ijerph17217980)
Supplement: Supplementary file 1 [file ijerph-17-07980-s001.pdf]

Supplementary Material for ‘Physical activity and psychosocial characteristics of the Peer leaders in the PLAN-A study – a latent class analysis’

Ruth Salway, Simon J. Sebire, Byron Tibbitts, Emily Sanderson, Rebecca Kandiayli, Kate Willis, Stephanie J MacNeill and Russell Jago

Additional tables

Table S1: Missing data for the whole study and among peer supporters

Table S2: Model fit and classification for models with 2-10 classes

Table S3: Item response probabilities and means for final 5-class model

Additional Figures

Figure S1: Distribution of classes and peer supporters between schools

MPlus code

Table S1. Missing data for the whole study and among peer supporters.

	All	Peer Supporters
	**N (%)**	**N (%)**
**Total**	**1558**	**357**
**Demographics**		
Family Affluence Scale	0 (0%)	0 (0%)
Free school meals	20 (1%)	3 (1%)
Ethnicity	5 (<1%)	1 (<1%)
**Self perception**		
Self-esteem	56 (4%)	14 (4%)
Self-efficacy	19 (1%)	6 (2%)
**Motivation**		
Autonomous	30 (2%)	6 (2%)
Controlled	14 (1%)	3 (1%)
Amotivation	17 (1%)	1 (<1%)
**PA among friends**		
Prevalence	8 (1%)	0 (<1%)
Importance	4 (<1%)	1 (<1%)
Acceptance	5 (<1%)	2 (1%)
PA peer support	18 (1%)	5 (1%)
**PA behaviours**		
Average weekday MVPA (mins)	127 (8%)	27 (8%)
Average weekday sedentary (mins)	127 (8%)	27 (8%)
Average weekday screen-viewing (mins)	35 (2%)	11 (3%)

PA: physical activity.

Table S2. Model fit and classification for models with 2-10 classes.

No. classes	BIC[1]	LMR[2]	BLRT[2]	Relative entropy	Average class classification probabilities[3]	% in smallest class
2	55039.6	<0.005	<0.005	0.813	0.94-0.95	47%
3	54519.4	<0.005	<0.005	0.785	0.89-0.91	21%
4	54373.1	0.01	<0.005	0.764	0.81-0.93	19%
5	54286.4	0.58	<0.005	0.750	0.81-0.91	12%
6	54232.4	0.58	<0.005	0.755	0.78-0.90	10%
7	54229.4	0.63	<0.005	0.745	0.68-0.91	10%
8	54237.8	0.62	<0.005	0.744	0.68-0.90	7%
9	Model unstable					
10	Model unstable					

[1] lower BIC indicates better model fit [2] *p*-value for test comparing the current number of classes to a model with one fewer classes [3] The model estimates the probability of being in each class for each participant. The average classification probability for each class is the average if each girl is assigned to the most likely class (i.e. the class with the highest probability). This column reports the minimum and maximum average classification probability across the classes; higher values indicate less uncertainty about class membership. BIC: the Bayesian Information Criterion, LMR: the Lo-Mendell-Rubin, BLRT: bootstrapped likelihood ratio tests.

Table S3. Item response probabilities and means for final 5-class model.

	Autonomous, Confident and High PA value	Relatively autonomous and Confident	Relatively controlled and High PA value	Relatively controlled, Low confidence and PA value	Amotivated, Low confidence and PA value
**Estimated proportion**	19%	18%	28%	23%	12%
**Self perception**					
Self-esteem[1]	0.89 (0.08)	0.81 (0.10)	0.66 (0.15)	0.48 (0.22)	0.50 (0.20)
Physical activity self-efficacy[1]	0.91 (0.08)	0.77 (0.12)	0.74 (0.13)	0.54 (0.19)	0.44 (0.21)
**Motivation**					
Autonomous[1]	0.88 (0.09)	0.57 (0.19)	0.73 (0.16)	0.52 (0.17)	0.23 (0.12)
Controlled [1]	0.35 (0.19)	0.16 (0.10)	0.44 (0.17)	0.42 (0.20)	0.15 (0.11)
Mod/high amotivation (>0.25)	6%	19%	26%	58%	76%
**PA among friends**					
**Peer Norm scales**					
High prevalence (3-6)	90%	69%	69%	25%	26%
High importance (3-6)	69%	23%	46%	21%	14%
High acceptance (3-6)	65%	37%	62%	29%	23%
Peer support[1]	0.66 (0.16)	0.50 (0.19)	0.53 (0.18)	0.35 (0.19)	0.28 (0.19)
**Behaviours**					
Average MVPA (mins)	53 (20)	52 (17)	54 (22)	48 (17)	48 (23)
Average sedentary (mins)	587 (81)	59 (90)	600 (93)	599 (90)	563 (108)
Average screen-viewing (mins)	237 (131)	375 (305)	338 (163)	507 (305)	553 (248)
**Mean characteristics in each class**					
% Peer supporter	43%	21%	26%	13%	7%
% meeting PA guidelines	22%	23%	28%	14%	24%
Family Affluence Score	7.5	6.8	6.9	6.6	6.1
% Free school meals	5%	9%	10%	14%	21%
Ethnicity: % non-white	8%	12%	13%	11%	13%

[1] Scaled to values between 0 and 1, for ease of comparison.

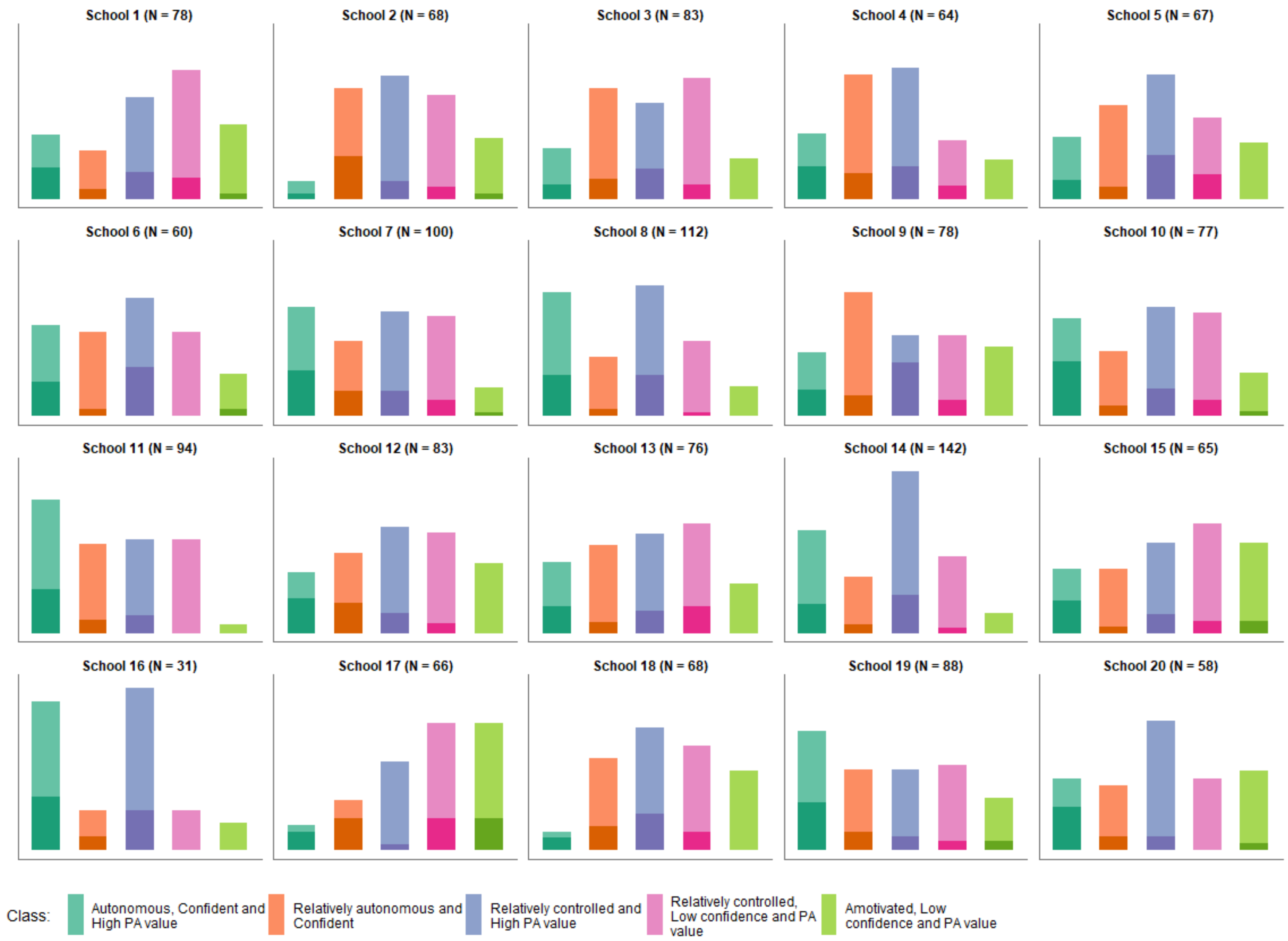

Figure S1. Distribution of classes and peer supporters between schools. Full bars indicate proportion of whole sample in each class, shaded areas indicate those who are peer supporters.

MPlus Code

```
TITLE:  Latent class analysis for peer supporter data:
      motivation, self, peer and PA behaviour
        Covariance between mvpa  & sed
        All variances & covariances different across classes
        Amotivation and Peer norms variables categorical

DATA:   FILE IS 'data for lca.csv';
        FORMAT IS FREE;
VARIABLE:
        NAMES ARE id school famaff fsm bame est eff
        auto cont amotcat pnprev pnimp pnacc  pasup
         mvpa sed sv  peer pa;
        USEVARIABLES est eff auto cont amotcat pnprev pnimp pnacc
        pasup mvpa sed sv;
        MISSING ARE ALL (-9);
        CATEGORICAL amotcat pnprev pnimp pnacc;
        IDVARIABLE = id;

      ! 5 latent classes with school-level clustering
        CLASSES = C(5);
        CLUSTER=school;

      ! estimate % of peer supporters, meeting guidelines, family
affluence,
      ! free school meals and BAME for each latent class
        AUXILIARY=(bch) peer pa famaff fsm bame;


ANALYSIS: TYPE = COMPLEX MIXTURE;
        STARTS 500 100;

MODEL:
                %OVERALL%
        ! allow covariance between MVPA & sed
      mvpa WITH sed (pacov);

        ! allow variances to differ in each class
                %C#1%
        est (v1a);
        eff (v1b);
        auto (v1c);
        cont (v1d);
        pasup (v1i);
        mvpa (v1j);
        sed (v1k);
        sv (v1l);

                %C#2%
        est (v2a);
        eff (v2b);
        auto (v2c);
        cont (v2d);
        pasup (v2i);
        mvpa (v2j);
        sed (v2k);
```

```
        sv (v2l);

                %C#3%
        est (v3a);
        eff (v3b);
        auto (v3c);
        cont (v3d);
        pasup (v3i);
        mvpa (v3j);
        sed (v3k);
        sv (v3l);

                %C#4%
        est (v4a);
        eff (v4b);
        auto (v4c);
        cont (v4d);
        mvpa (v4j);
        sed (v4k);
        sv (v4l);

                %C#5%
        est (v5a);
        eff (v5b);
        auto (v5c);
        cont (v5d);
        pasup (v5i);
        mvpa (v5j);
        sed (v5k);
        sv (v5l);


! save most likely class
SAVEDATA:
    file is "basic classes.csv";
    save=cprob;
    format=free;
    missflag=-9;
```
